# Risk factors for infection after hysterectomy for benign disease

**DOI:** 10.1111/aogs.70362

**Published:** 2026-10-05

**Authors:** Sophia Ehrström, Karin Källén, Andrea Stuart

**Affiliations:** ^1^ Division of Obstetrics and Gynecology, Department of Clinical Sciences Karolinska Institutet, Danderyd Hospital Stockholm Sweden; ^2^ Centre for Reproductive Epidemiology Lund University Lund Sweden; ^3^ Department of Obstetrics and Gynecology Institution of Clinical Sciences, Helsingborg Hospital Helsingborg Sweden; ^4^ Lund University Lund Sweden

**Keywords:** depression, endometriosis, hysterectomy, immunoregulatory drugs, perioperative complication, population‐based study, postoperative infection, psycholeptics

## Abstract

**Introduction:**

Our objective was to identify risk factors for infections following hysterectomy for benign disease.

**Material and Methods:**

This population‐based cohort study included hysterectomies recorded in the Swedish National Quality Register for Gynecological Surgery (GynOP) between 2005 and 2022. Data were linked with the Swedish Prescribed Drug Register, the National Patient Register, and Statistics Sweden. Postoperative infection was defined as a dispensed systemic antibiotic prescription within 21 days postoperatively (i.e., not local antibiotics or injectables). Crude and adjusted risk ratios (aRRs) were estimated using modified Poisson regression, adjusting for preoperative medications, demographic and perioperative factors.

**Results:**

Among 50 601 hysterectomies, 7153 women (14.1%) had a dispensed prescription of antibiotics postoperatively. Younger age, antidepressant or psycholeptic use, and previous abdominal or cesarean surgery were associated with increased risk of dispensed antibiotics. Endometriosis (aRR 1.30, 95% CI 1.06–1.58), preoperative use of immunoregulatory drugs (aRR 1.48, 95% CI 1.09–2.00), and Body Mass Index (BMI) ≥40 kg/m^2^ (aRR 1.42, 95% CI 1.20–1.68) were independent risk factors. Perioperative complication was the strongest predictor (aRR 2.19, 95% CI 1.97–2.44) for dispensed antibiotics.

**Conclusions:**

Prescribed and dispensed antibiotics after benign hysterectomy are common and influenced by several patient‐ and surgical factors. Identification of high‐risk patients such as women with high BMI or using immunomodulating drugs, patients with endometriosis or previous abdominal surgery may support individualized preventive strategies and improve postoperative outcomes.

## Graphical abstract

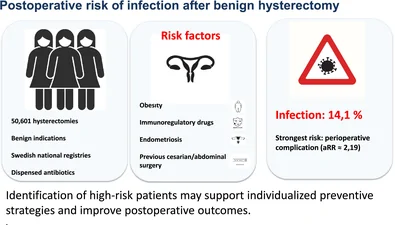

**Abbreviations**
AH: abdominal hysterectomy
aRR/aRRs: adjusted risk ratio(s)
BMI: Body Mass Index
CI: confidence interval
CS: cesarean section
GynOP: Swedish National Quality Register for Gynecological Surgery
ICD/ICD‐10: International Classification of Diseases
LAVH: laparoscopic‐assisted vaginal hysterectomy
RALH: robot‐assisted laparoscopic hysterectomy
RR: risk ratio
SSI: surgical site infection
TLH: total laparoscopic hysterectomy
VH: vaginal hysterectomy

**Key message**

Pre‐ and perioperative risk factors, including high BMI, immunomodulatory treatment, endometriosis, younger age, and previous abdominal or cesarean surgery, can be integrated into clinical risk assessment to identify women at increased risk of postoperative infectious morbidity and to guide individualized preventive strategies after hysterectomy.

## 1 INTRODUCTION

Hysterectomy is one of the most common surgeries performed in women aged 30–60 years. During the last 5 years, 3500–4000 hysterectomies are performed for benign indications in Sweden; the corresponding rate in the United States is 590 000.^1^, ^2^, ^3^

Postoperative complications vary based on route of surgery and surgical technique. Infectious complications after hysterectomy are the most common, ranging from 10.5% for abdominal hysterectomy (AH) to 13.0% for vaginal hysterectomy (VH) and 9.0% for total laparoscopic hysterectomy (TLH).^4^, ^5^, ^6^ Known risk factors for postoperative infection include AH, obesity, prolonged operative time, diabetes, and smoking.^7^, ^8^, ^9^, ^10^, ^11^ Most studies focus on surgical‐site infections (SSI) and therefore underestimate the total burden of postoperative infectious morbidity. Furthermore, there is a paucity of data on how preoperative demographics and medical factors covary with perioperative factors in shaping postoperative infection risk.

The aim of the study was to evaluate established and previously unidentified risk factors for postoperative infection by incorporating preoperative medical treatment data to perioperative data to identify patient groups at increased risk. Postoperative infection was assessed using dispensation of prescribed antibiotics.

## 2 MATERIAL AND METHODS

Data was merged from four Swedish National based registries 2005–2022. Women who had a hysterectomy, with or without concomitant adnexal surgery, were identified from The Swedish National Quality Register for Gynecological Surgery (GynOP).^3^ Postoperative infection was defined as dispensed antibiotics within 21 days after surgery, identified through the national prescription drug register. A 21‐day postoperative window was chosen to ensure that antibiotic treatment was temporally related to the hysterectomy, as infections attributable to surgery typically occur within the first three postoperative weeks. Only prescribed and dispensed antibiotics were included in the outcome. Routine postoperative antibiotic use is not practiced in Sweden for prophylaxis. Therefore, postoperative antibiotic prescription is a valid surrogate marker for infection.

Surgical techniques include abdominal hysterectomy (AH) and the minimally invasive surgeries; vaginal hysterectomy (VH), laparoscopic assisted vaginal hysterectomy (LAVH), total laparoscopic hysterectomy (TLH), and robot assisted laparoscopic hysterectomy (RALH). Recently, vaginal natural orifice transluminal endoscopic surgery (vNOTES) hysterectomy has been introduced as a new minimally invasive method; however, Swedish data concerning the method were not available in this data set. Subtotal hysterectomy (which is performed in small numbers in Sweden) was never included in the analysis, only data regarding total hysterectomies were used in the analysis.

The personal identification number was used to retrieve data from three national Swedish registries: Swedish Prescribed Drug Register (PDR), Statistics Sweden, and the Swedish National Patient Register (NPR).

GynOP contains preoperative, intraoperative, and postoperative information regarding gynecological surgery since 1997. The data registration rate is high regarding perioperative data, and the register provides complementary data with high accuracy.^12^, ^13^ Approximately 48–50 clinics in Sweden report benign hysterectomies to the national register each year, corresponding to a coverage of 90%–96%.

Demographic variables are filled in at the time of surgery (age, Body Mass Index (BMI), smoking, previous abdominal surgery including laparoscopy and laparotomy, and previous cesarean section (CS)), type of surgery (hysterectomy and/or adnexal surgery), primary incision (abdominal, laparoscopic, robotic, vaginal), conversion to laparotomy and indication for surgery. Postoperatively, the surgeon registers surgical data such as uterus weight, blood loss, and duration of surgery. A tick box description of perioperative complications found in the GynOP Register was used. No further specification of type of perioperative complication is available in our data from the GynOP register. For further specification of the type of complication, data regarding the Swedish National Patient Registry was used.

The Swedish National Patient Register contains data on patients admitted to all Swedish hospitals since 1987; date of admission, date of discharge, and diagnoses and procedural codes registered for each admission. Codes are classified according to the International Classification of Diseases, 10th Revision codes. ICD‐10 codes for complications as specified below were collected from the Patient Register. Indications for hysterectomy were defined as follows: endometriosis N80, prolapse N81, dysmenorrhea N94, cervical dysplasia N87, endometrial hyperplasia N85, menometrorrhagia N92, postmenopausal bleeding N95, and myoma D25. Women with dysmenorrhea had no documented endometriosis diagnosis. Indication for the hysterectomy was found from both the GynOP Register and the Patient Register.

Intraoperative complications were defined as follows: urinary bladder injury (ICD code S37.2, procedural code suture of urinary bladder: KCH00, KCH96), ureter injury (ICD code S37.1, procedural code suture of ureter: KBH00, KBH96), bowel injury (ICD code S36.4–66, procedural code suture of rectum: JGA60), accidental puncture and laceration during a procedure (ICD code T81), not elsewhere classified (ICD code T81.2) or vascular complications following a procedure, not elsewhere classified (ICD code T81.7). ICD and procedural codes were identified from the Patient Register.

The Prescribed Drug Register was established in 2005 and is maintained by the Swedish National Board of Health and Welfare. It contains data on all prescribed drugs dispensed at pharmacies in Sweden. Pharmaceutical consumption is classified according to the Anatomic Therapeutic Chemical Classification (ATC). Prescriptions of drugs were extracted to identify preexisting comorbidities such as cardiovascular disease or diabetes (3 months before surgery) and to assess postoperative antibiotic dispension (1–21 days after surgery). The 3‐week postoperative window was selected to reduce the risk of capturing antibiotic use unrelated to surgery‐associated infections. Postoperative infection was defined as dispensed prescription of antibiotics 1–21 days postop (ATC code J01 found in the Prescribed Drug Register).

Data regarding drug consumption 3 months before surgery was extracted from The Prescribed Drug Register; A10A insulin, A10B oral antidiabetic agents, B01 antithrombotic agents, C01 cardiac therapy, C02 antihypertensives, C03 diuretics, C04 peripheral vasodilators, C05 vasoprotectives, C07 beta blocking agents, C08 calcium channel blockers, C09 agents acting on the renin‐angiotensin system, C10 lipid modifying agents, N05 psycholeptics, N06A antidepressants, N06B psychostimulants, H02AB glucocorticosteroids, L01XC monoclonal antibodies, L03 immunostimulant drugs, L04 immunosuppressing drugs.

Statistics Sweden is a government agency that produces official statistics. Data regarding country of birth was obtained from Statistics Sweden.

Preoperative antibiotics were most often administered orally in accordance with national recommendations by the Swedish strategic program against antibiotic resistance (STRAMA).^14^ Sweden has a recommendation of a rather restricted use of antibiotics compared to other countries.

### 2.1 Statistical analysis

Crude (Model 1) and adjusted Risk Ratios (aRRs) (Model 2 and 3) for dispensed antibiotics were obtained using modified Poisson regression analyses. Adjustments were made in two steps. In the first multivariable model (Model 2) preoperative demographics were included. Those were: age (classes: <50, 50–59, 60–69, and ≥70 years), medications (diabetic drugs, cardiac/antihypertensive drugs, psycholeptic stimulators, antidepressants, immunoregulatory drugs, or none of these), reproductive history (parity, vaginal births, cesarean sections), BMI (classes: <18.5, 18.5 to 24.9, 25.0 to 29.9, 30.0 to 34.9, 35.0 to 39.9 and ≥40 kg/m^2^), previous abdominal surgery, country of birth (reference is Sweden or another Nordic country, i.e.Sweden, Norway, Denmark, Finland and Island), Europe outside the Nordic countries, Non‐ European country, mode of hysterectomy (abdominal, robotic, vaginal, laparoscopic), uterus weight (≤300, 301–500, 501–1000, or ≥1000 g), indication for hysterectomy (menorrhagia/pain/dysmenorrhea, prolapse, endometriosis, postmenopausal bleeding, cervical dysplasia, or other indication). In the second step (Model 3), adjustments were also made for perioperative factors. These were: surgical time (linear continuous variable, per 30 min increments), any perioperative complications (yes, no), conversions to laparotomy (yes, no) or blood loss (classes <500, 500–999, ≥1000 mL).

Model 2 represents the primary multivariable model, adjusted for preoperative demographic and clinical factors. This model reflects the overall association between patient‐ and procedure‐related characteristics and dispensed antibiotics. Model 3 additionally includes perioperative events; complications, conversions to laparotomy, surgical time, and blood loss. Because these events occur during surgery, Model 3 should not be interpreted as a final or fully adjusted model. Instead, it is presented to illustrate how much of the association may be explained by perioperative events. By comparing Model 2 with Model 3, we show the extent to which perioperative complications influence the risk estimates.

Only records without missing values were included in the analyses. Statistical analyses were performed using IBM SPSS Statistics, version 27.0.

## 3 RESULTS

Eighty‐four thousand eight hundred ninety‐five hysterectomies were identified in the GynOP register. A complete‐case approach was used. Variables with missing data included operation type (0.6%), operation time (1.1%), blood loss (2.9%), BMI (26.2%), and uterus weight (18.3%). In total, 34 294 procedures (40.4%) had missing data in at least one of these variables and were therefore excluded from the analyses, resulting in 50 601 complete cases (59.6%) (Table S1). We examined patterns of missingness and found no meaningful differences by calendar period, surgical route, or hospital.

Complete data regarding 50 601 hysterectomies performed 2005–2022 was extracted from the Swedish GynOP Register. Table 1 presents background characteristics of the study population, presenting row percentages within each subgroup. In the total cohort, 14.1% had dispensed antibiotics postoperatively. Higher rates were observed among women under 50 years of age (15.4%) compared with those over 70 (11.3%), among women with two previous cesarean sections (19.1%), with surgical duration exceeding 120 min (17.7%), with BMI over 35 and 40 (20.3% and 27.1%, respectively), and among women with endometriosis (23.6%).

**TABLE 1:** Percentage of postoperatively dispensed antibiotics after hysterectomy according to surgical and patient demographics in a Swedish population cohort study, 2005–2022.

	Total	Dispensing of antibiotics (J01) 1–21 days after operation		No postoperative antibiotics	
	*N*	*n*	(%)	*n*	(%)
All	50 601	7153	(14.1)	43 448	(85.9)
Mode of hysterectomy					
Abdominal	25 022	3525	(14.1)	21 497	(85.9)
Laparoscopic	7187	1097	(15.3)	6090	(84.7)
Robotic	5510	847	(15.4)	4663	(84.6)
Vaginal	12 882	1684	(13.1)	11 198	(86.9)
Uterus weight (g)					
≤300	32 712	4661	(14.2)	28 051	(85.8)
301–500	7128	1014	(14.2)	6114	(85.8)
501–1000	7543	1027	(13.6)	6516	(86.4)
>1000	3218	451	(14.0)	2767	(86.0)
Surgical duration (min)					
<45	3413	425	(12.5)	2988	(87.5)
45–59	5819	719	(12.4)	5100	(87.6)
60–89	15 845	1992	(12.6)	13 853	(87.4)
90–120	12 605	1727	(13.7)	10 878	(86.3)
≥120	12 917	2290	(17.7)	10 627	(82.3)
Perioperative complications	1846	578	(31.3)	1268	(68.7)
Blood loss (mL)					
<100	19 283	2430	(12.6)	16 853	(87.4)
100–499	26 219	3757	(14.3)	22 462	(85.7)
500–999	4020	688	(17.1)	3332	(82.9)
≥1000	1079	278	(25.8)	801	(74.2)
Conversion laparotomy	985	177	(18.0)	808	(82.0)
Previous abdominal surgery	8124	1458	(17.9)	6666	(82.1)
Reproductive history					
0‐parous	4739	652	(13.8)	4087	(86.2)
Para, no CS	37 166	4976	(13.4)	32 190	(86.6)
1 previous CS	5594	933	(16.7)	4661	(83.3)
>1 previous CS	3102	592	(19.1)	2510	(80.9)
BMI (kg/m^2^)					
<18.5	414	54	(13.0)	360	(87.0)
18.5–24.9	21 425	2814	(13.1)	18 611	(86.9)
25.0–29.9	17 887	2376	(13.3)	15 511	(86.7)
30.0–34.9	7831	1242	(15.9)	6589	(84.1)
35.0–39.9	2301	466	(20.3)	1835	(79.7)
≥40	743	201	(27.1)	542	(72.9)
Age (years)					
<50	30 452	4683	(15.4)	25 769	(84.6)
50–59	12 496	1633	(13.1)	10 863	(86.9)
60–69	4431	474	(10.7)	3957	(89.3)
≥70	3222	363	(11.3)	2859	(88.7)
Smoking	2539	395	(15.6)	2144	(84.4)
Preoperative medications					
Diabetic drugs	520	85	(16.3)	435	(83.7)
Cardiac/Hypertensive drugs	4990	829	(16.6)	4161	(83.4)
Psycholeptics or stimulators	4088	829	(20.3)	3259	(79.7)
Antidepressants	2824	564	(20.0)	2260	(80.0)
Immuno‐regulation drugs	195	51	(26.2)	144	(73.8)
Need of language interpreter	523	82	(15.7)	441	(84.3)
Region in Sweden					
Stockholm	4529	808	(17.8)	3721	(82.2)
Central	10 104	1437	(14.2)	8667	(85.8)
South East	7266	937	(12.9)	6329	(87.1)
Southern	11 519	1491	(12.9)	10 028	(87.1)
Western	10 894	1634	(15.0)	9260	(85.0)
Northern	6289	846	(13.5)	5443	(86.5)
Indication for surgery					
Prolapse	4572	495	(10.8)	4077	(89.2)
Endometriosis	474	112	(23.6)	362	(76.4)
Pain/pressure	5311	781	(14.7)	4530	(85.3)
Postmenopausal bleeding	1196	144	(12.0)	1052	(88.0)
Cervical dysplasia	2872	294	(10.2)	2578	(89.8)
Menorraghia	23 240	3502	(15.1)	19 738	(84.9)
Dysmenorrhea	2049	348	(17.0)	1701	(83.0)
Ovarian mass	2505	339	(13.5)	2166	(86.5)
Other	5264	691	(13.1)	4573	(86.9)
Not specified	3118	447	(14.3)	2671	(85.7)

Table 2 presents the crude and multivariable analysis of risk factors for dispensed antibiotics. Presurgical factors and perioperative events such as bleeding, complications, conversion to laparotomy, and surgical time are shown in separate columns, and these variables are incorporated stepwise across the three models.

**TABLE 2:** Univariate and multivariable analysis. Risk factors for dispensed antibiotics after benign hysterectomy after adjusting for demographic data in a first step, and surgical data in a second step.

Factor	Model 1 Crude Univariate		Multivariable model 2 preoperative demographics		Multivariable model 3 including demographic and perioperative complications/events	
	RR	95%CI	aRR^a^	95%CI	aRR^b^	95%CI
Year of operation						
2005–2010	1.04	0.97–1.13	1.12	1.02–1.23	1.08	0.99–1.19
2011–2016	0.94	0.88–1.01	1.02	0.94–1.09	1.00	0.93–1.07
2017–2022	1.00	Reference	1.00	Reference	1.00	Reference
Age (years)						
<50	1.00	Reference	1.00	Reference	1.00	Reference
50–59	0.84	0.78–0.90	0.87	0.82–0.94	0.87	0.82–0.94
60–69	0.64	0.58–0.72	0.73	0.65–0.83	0.73	0.65–0.82
≥70	0.68	0.61–0.76	0.76	0.66–0.88	0.76	0.66–0.87
Medications						
Diabetic drugs	0.95	0.74–1.21	0.96	0.75–1.23	0.97	0.76–1.25
Cardiac/hypertensive drugs	1.15	1.06–1.24	1.24	1.14–1.35	1.24	1.14–1.35
Psycholeptic stimulators	1.33	1.22–1.44	1.29	1.19–1.41	1.29	1.19–1.41
Antidepressants	1.22	1.10–1.36	1.16	1.05–1.28	1.15	1.04–1.28
Immuno‐regulation drugs	1.51	1.10–2.08	1.52	1.11–2.07	1.48	1.09–2.00
None of the above drugs	1.00	Reference	1.00	Reference	1.00	Reference
Reproductive history						
Nulliparous	1.06	0.96–1.16	0.94	0.85–1.04	0.94	0.85–1.03
Parous, no CS	1.00	Reference	1.00	Reference	1.00	Reference
Parous, previous CS	1.25	1.17–1.33	1.11	1.04–1.19	1.09	1.02–1.17
Previous other abdominal surgery	1.29	1.21–1.38	1.24	1.16–1.33	1.24	1.16–1.32
Body Mass Index (kg/m^2^)						
<18.5	1.12	0.85–1.48	1.12	0.85–1.48	1.12	0.85–1.47
18.5–24.9	1.00	Reference	1.00	Reference	1.00	Reference
25.0–29.9	0.96	0.90–1.02	0.94	0.89–1.01	0.94	0.88–1.00
30.0–34.9	1.04	0.96–1.12	0.98	0.91–1.06	0.96	0.88–1.04
35.0–39.9	1.32	1.17–1.48	1.21	1.07–1.36	1.15	1.02–1.29
≥40	1.68	1.42–1.98	1.52	1.28–1.80	1.42	1.20–1.68
Country of birth						
Nordic countries^a^	1.00	Reference		Reference		Reference
European, non‐Nordic	0.96	0.82–1.12	0.99	0.84–1.15	0.99	0.85–1.16
Non‐European	1.22	1.03–1.44	1.12	0.95–1.33	1.11	0.93–1.31
Mode of hysterectomy						
Abdominal	0.97	0.89–1.05	0.92	0.84–1.02	0.96	0.86–1.05
Robotic	1.01	0.93–1.09	0.98	0.90–1.06	1.02	0.94–1.11
Vaginal	0.86	0.80–0.92	0.92	0.85–1.00	0.98	0.90–1.07
Laparoscopic	1.00	Reference	1.00	Reference	1.00	Reference
Uterus weight (g)						
≤300	1.00	Reference	1.00	Reference	1.00	Reference
301–500	1.11	1.02–1.20	1.04	0.96–1.14	1.01	0.93–1.10
501–1000	1.07	0.98–1.16	1.03	0.93–1.14	0.97	0.88–1.08
>1000	1.22	1.08–1.37	1.18	1.02–1.35	1.06	0.92–1.22
Indication for hysterectomy						
Menorrhagia/pain/dysmenorrhea	1.00	Reference	1.00	Reference	1.00	Reference
Prolapse	0.68	0.62–0.75	0.88	0.78–0.98	0.88	0.78–0.99
Endometriosis	1.62	1.34–1.96	1.50	1.24–1.83	1.30	1.06–1.58
Postmenopausal bleeding	0.80	0.66–0.97	0.92	0.76–1.13	0.93	0.76–1.14
Cervical dysplasia	0.63	0.55–0.71	0.69	0.60–0.79	0.69	0.60–0.78
Other	0.87	0.81–0.94	0.93	0.86–1.01	0.92	0.86–1.00
Surgical time (per 30 min increment)	1.09	1.08–1.11	–	–	1.04	1.02–1.06
Perioperative complications	2.49	2.28–2.72	–	–	2.19	1.97–2.44
Blood loss (mL)			–	–		
<500	1.00	Reference	–	–	1.00	Reference
500–999	1.32	1.19–1.47	–	–	1.03	0.92–1.16
≥1000	1.96	1.68–2.28	–	–	1.01	0.84–1.20
Conversion to laparotomy	1.28	1.11–1.47	–	–	0.91	0.79–1.05

^a^ Nordic countries = Sweden, Norway, Denmark, Finland, Iceland.

In Model 3, the risk of dispensed antibiotics decreased with increasing age, with women aged ≥70 having a lower risk compared with women under 50 (aRR 0.76, 95% CI 0.66–0.87). Women operated for prolapse (aRR 0.88, 95% CI 0.78–0.99) or dysplasia (aRR 0.69, 95% CI 0.60–0.78) had lower risks compared with those operated for menorrhagia or pain. Increasing BMI was associated with a higher risk of dispensed antibiotics, particularly BMI ≥40 (aRR 1.42, 95% CI 1.20–1.68). Endometriosis was associated with a higher risk of dispensed antibiotics. The crude estimate (RR 1.62) attenuated after adjustment (Model 2: 1.50; Model 3: 1.30). The risk remained after adjustment for perioperative events, suggesting that women with endometriosis have an independently elevated risk of postoperative dispensed antibiotics. Women who had dispensed antihypertensive drugs, cardiac drugs, antidepressants, antipsychotics, or immunoregulatory drugs in the 3 months before surgery had significantly higher risks of dispensed antibiotics; immunoregulatory drugs increased the risk by approximately 50% (aRR 1.48, 95% CI 1.09–2.00). Previous CS (aRR 1.09, 95% CI 1.02–1.17) and previous abdominal surgery (aRR 1.24, 95% CI 1.16–1.32) were also associated with increased risk.

In the crude model, VH showed a 14% lower risk of dispensed antibiotics compared with laparoscopic hysterectomy, but this association was not present in Model 3 after adjustment for perioperative complications. Perioperative complications had the strongest impact on the risk of antibiotic dispension (aRR 2.19, 95% CI 1.97–2.44). Bleeding >1000 mL and conversion to laparotomy increased the risk in the univariable model but not in Model 3. Conversion to laparotomy increased the risk in the univariable analysis (aRR 1.28, 95% CI 1.11–1.47), but this association did not remain after adjustment.

In Model 3, AH did not affect the risk of postoperative dispensed antibiotics. Uterine weight showed increased risk of dispensed antibiotics in the crude model and in Model 2. When perioperative factors were taken into account (Model 3), the association disappeared, indicating that it is the complicating perioperative events rather than uterus size per se that increase the risk of postoperative dispensed antibiotics.

## 4 DISCUSSION

We studied a large population‐based cohort of 50 601 hysterectomies with data registered in four validated Swedish national quality registries. Our results showed that the rate of dispensed antibiotics increased with previously known factors such as increasing BMI, perioperative complications, and endometriosis. The study adds new data that infection rates are increased in younger women, in patients with previous cesarean or previous abdominal surgery, and in women using immunomodulating drugs, psycholeptics, cardiovascular, or antidepressant drugs 3 months prior to surgery. The risk was lower in patients undergoing hysterectomy for dysplasia or prolapse, and in older women.

A strength with our study was the use of population‐based data from four different registries in a large cohort of women who had undergone a hysterectomy, giving a comprehensive risk assessment with both social, perioperative, and medical risk factors. Detailed risk factors, such as the use of antidepressants or immunomodulating drugs, could be analyzed. Another strength of the study is that dispensed prescriptions of antibiotics were used to define infection. Dispensed antibiotics offer a clinically meaningful indicator of postoperative infection, reflecting real‐world treatment decisions for confirmed or suspected infections across both hospital and primary care settings. There is no formal validation of the exact proportion of postoperative infections reported in the surgeon‐reported tick‐box variable in GynOp. We believe the registry lacks sufficient information to reliably identify postoperative infections. Many postoperative infections are managed in primary care and are therefore not reported back to the operating clinic. It is also not feasible to identify all infection‐related ICD codes in the National Patient Register, as postoperative infections can lead to a wide range of diagnostic codes. For these reasons, we chose dispensed systemic (not local or injectable) antibiotics as the outcome, as it provides a clinically relevant and clearly defined measure of treated infection. Pharmacy dispensing data has full national completeness and captures infections treated both in hospital and in primary care.

A weakness of the study is that data, although recorded prospectively, were analyzed retrospectively. We compared missingness of data by calculating the proportion of missing values within each calendar period and surgical route and checking whether these proportions differed across strata. The proportions were essentially identical. Center‐level clustering was not accounted for, and the estimates with confidence intervals close to unity should be interpreted with this in mind. Antibiotics prescribed for other indications than surgery‐related infections were not possible to exclude from the analysis, but we believe that within the first 21 days after surgery, these patients should be few and not alter the results. ASA classification was not available, but we believe that the information regarding BMI in combination with drugs prescribed 3months before the surgery catches morbidity in the patients in a broader view and takes the analysis a step further in classifying the group of drugs that are associated with the highest risk of dispensed antibiotics.

The results suggest the use of immunoregulatory drugs within 3months preoperatively as a main risk factor, and not classic diabetic drugs (usually indicating a higher ASA‐group). In the GynOP Register, 195 patients taking immunomodulatory drugs underwent a hysterectomy, and almost 30% of the patients had dispensed antibiotics postoperatively. A Cochrane review assessing the risk of immunomodulatory agents on postoperative infection risk within 30 days of surgery showed increased overall infectious complications in patients who received anti‐TNF agents (OR 1.60; 95% CI 1.20 to 2.13). The review concluded that the impact of these medications on postoperative infectious complications is uncertain, and no firm conclusions could be drawn regarding their safety in the perioperative period.^15^ The Swedish Federation of Rheumatology has guidelines for cessation of immunomodulating drugs prior to surgery for orthopedic surgery, and these guidelines are commonly extrapolated for patients undergoing hysterectomy.^16^ Further research is needed to assess if patients using immunomodulating drugs prior to hysterectomy would benefit from cessation of immunomodulating drugs or different antibiotic prophylaxis.

Interestingly, preoperative treatment with antipsychotics or antidepressants was also associated with a higher risk of dispensed antibiotics compared to antidiabetic drugs, which has not been reported previously. We hypothesize that the suppression of the immune system in depressive disorders may expose the patients to increased rates of postoperative infections, as has been suggested in a previous study.^17^ The increased antibiotic dispension may also reflect differences in healthcare‐seeking behavior among patients taking antipsychotics or antidepressants.

Despite advancements and the widespread adoption of minimally invasive techniques over the past 15 years, infection rates do not appear to have decreased substantially according to the Swedish GynOP register.^18^ When dividing the cohort in three time periods (Table 2), no overall significant change in dispensed antibiotics was evident. Robotic, vaginal, and laparoscopic techniques offer benefits such as faster recovery and reduced pain postoperatively compared to laparotomy.^19^, ^20^, ^21^, ^22^, ^23^, ^24^, ^25^, ^26^ The adoption of robotic and laparoscopic surgery has risen every year the last decade. Simultaneously, the frequency of vaginal hysterectomies has declined.^3^, ^27^ VH has previously reported lower or similar infection rates compared to laparoscopic and robotic approaches, and lower rates of complications and risk for fever and urinary tract infections, compared to laparotomy.^4^, ^25^, ^26^

From a resource perspective VH is, based on solid scientific evidence, recommended as the first line minimally invasive surgical option due to its low cost, short operation time and fast recovery.^24^, ^25^, ^26^, ^27^ However, over the past decade, the use of this method has been halved (similar figures can be seen in other high‐income countries), and opportunities for structured training have been significantly reduced in Sweden, as in other countries.^27^ Although minimally invasive surgery reduces pain, recovery time, and hospital stay, our results indicate that minimally invasive surgery alone does not reduce clinically treated postoperative infections. This suggests that infection risk is driven more by patient‐related factors and surgical complexity than by surgical approach.

The risk of dispensed antibiotics was substantially higher if the woman had previously undergone a cesarean section or previous abdominal surgery. Previous abdominal surgery can complicate the hysterectomy due to adhesions or distorted anatomy, as previously reported.^28^, ^29^ As CS rates continue to increase worldwide, the proportion of women undergoing hysterectomy with prior CS will rise, making this risk factor increasingly relevant for surgical planning and infection prevention.

Previous reports have suggested an association between longer duration of surgery in RALH and laparoscopy as a risk factor for infection.^30^ In our study, we could confirm that longer operative time showed a slightly elevated risk for infection postoperatively. Based on an aRR of 1.04 per 30‐min increment, a hysterectomy lasting 3 h carries approximately 17% higher odds of postoperative antibiotics compared with a one‐hour procedure, even after adjusting for the presence of perioperative complications. Women with perioperative complications had the highest risk of dispensed antibiotics. Although bleeding >1000 mL and conversion to laparotomy increased the risk in univariable analyses, these associations were not present in Model 3, suggesting that the increased risk is primarily attributable to the overall presence of a perioperative complication rather than the individual components of bleeding or conversion to laparotomy.

Patients younger than 40 had an 11% higher risk of infection (not shown in Table) than women between 40 and 50, and above 50 years of age the risk decreased continuously. Possible explanations could be that younger women are more sexually active with a different bacterial flora/bacterial vaginosis, increasing the risk of infection compared to the bacterial flora in women with lower estrogen levels.

Endometriosis as an indication for hysterectomy presented a higher risk of postoperative dispensed antibiotics, putatively due to the presence of extensive adhesive tissue in need of lysis and potential complications involving adjacent organs, which can increase vulnerability to infections. Our results are in line with another retrospective large cohort with 29 742 patients, comparing risk of infection after hysterectomy due to endometriosis or other benign indication, specifically reporting higher prevalence of deeper surgical site/organ space infection in patients with endometriosis.^31^

Several factors have previously been identified that reduce the risk of infection. Surgery site infection (SSI) is less frequent in minimally invasive surgery, compared to abdominal technique.^32^ Preoperatively, skin preparation with chlorhexidine‐alcohol decreases the risk of superficial SSI.^33^, ^34^, ^35^ Diabetes should be well controlled to reduce postoperative infections.^36^ Smoking cessation has been proposed,^37^ as well as hair shortening,^38^ vaginal cleansing and preoperative treatment of bacterial vaginosis and trichomonas.^33^ Intraoperatively, reduction of operative time and bleeding has been advocated to reduce the risk of infection. Repeated antibiotic prophylaxis has been suggested if more than a 2 h duration of surgery or bleeding more than 1500 mL^35^ has occurred. Urinary tract infections are reduced by removing the urinary catheter quickly (6 h) after surgery.^39^, ^40^

Integrating preoperative medication, demographic factors, and perioperative data are important for identifying women at higher risk of infectious complications, and future randomized controlled studies are needed to elucidate possible models of antibiotic prophylaxis to reduce postoperative infections.

## 5 CONCLUSION

Our study highlights important risk factors for dispensed antibiotics following hysterectomy, emphasizing the need for vigilance pre‐ and postoperatively. Women on immunomodulatory drugs, with BMI >40, or with endometriosis are at the highest risk. Integrating these risk factors into preoperative risk assessment may help identify women at increased likelihood of postoperative infectious morbidity and guide tailored prophylactic and perioperative strategies to reduce infection rates.

## AUTHOR CONTRIBUTIONS

All authors have contributed to the conception and design of the study, as well as analysis and interpretation of data. SE and AS drafted the article and KK, SE and AS revised it. All authors approved the final version of the article.

## FUNDING INFORMATION

The study was supported by grants from the Stig and Ragna Gorthon Foundation. The funders had no role in the study design, data analysis, data interpretation, or writing the report.

## CONFLICT OF INTEREST STATEMENT

The authors have no conflict of interest to declare.

## ETHICS STATEMENT

Ethical permission (number 2022‐01489‐01) was obtained from the Swedish national ethics board on April 13, 2022.

## Supporting information


**Table S1.** Exclusions (for flowchart infection after benign hysterectomy).

## Data Availability

The data that support the findings of this study are available on request from the corresponding author. The data are not publicly available due to privacy or ethical restrictions.
