# Supplementary material for: Risk factors for infection after hysterectomy for benign disease
Source: Acta Obstet Gynecol Scand. 2026 Oct 5:10.1111/aogs.70362. Online ahead of print. doi: 10.1111/aogs.70362 (PMC13639321; doi:10.1111/aogs.70362)
Supplement: Supplementary file 1 [file AOGS-9999-0-s001.docx]

| Supplemental Information Table S1. | | | |
| --- | --- | --- | --- |
|  | n | ( % ) |  |
| Operation type | 517 | ( 0.6) |  |
| Operation time | 906 | ( 1.1) |  |
| Blood loss | 2455 | ( 2.9) |  |
| BMI | 22236 | (26.2) |  |
| Uterus weight | 15530 | (18.3) |  |
| Educational level | 556 | ( 0.7) |  |
| Any of these exclusions | 34294 | (40.4) |  |
| Included | 50601 | (59.6) |  |
| Total | 84895 | 100 |  |

S1: Exclusions (for flowchart infection after benign hysterectomy)
